# Perforated Meckel’s Diverticulum and Adhesive Intestinal Obstruction in a Preterm Neonate: A Case Report

**DOI:** 10.7759/cureus.56208

**Published:** 2024-03-15

**Authors:** Salman M. Ghazwani, Safwan Ahmad Khan, Atheer Y.O. Hakami, Afnan Alamer, Bashair A. Medkhali

**Affiliations:** 1 Faculty of Medicine, Jazan University, Jazan, SAU; 2 Pediatric Surgery, King Fahad Hospital, Jazan, SAU; 3 Pediatric Surgery, Jazan University, Jazan, SAU; 4 College of Medicine, Jazan University, Jazan, SAU

**Keywords:** neonatal intestinal obstruction, neonatal adhesive intestinal obstruction, neonatal pneumoperitoneum, perforated meckel's diverticulum, neonatal perforation

## Abstract

Perforated bowel and adhesive intestinal obstruction are common indications for emergency surgical intervention in a preterm neonate. The initial approach to managing perforation involves either peritoneal drain insertion or formal laparotomy. Once a neonate presents with complete bowel obstruction, prompt abdominal exploration becomes crucial. One prevalent cause of bowel obstruction in this population is adhesions resulting from previous surgeries. This report details the case of a preterm, extremely low birth weight neonate experiencing pneumoperitoneum, initially managed with an intraperitoneal drain. Despite temporary improvement, the infant developed recurrent pneumoperitoneum, necessitating formal exploratory laparotomy. Approximately one month post-surgery, the baby encountered complete bowel obstruction due to adhesive intestinal obstruction, requiring a second exploratory laparotomy. The child survived both surgical interventions and is thriving at follow-up. Our findings suggest that in select cases, intraperitoneal drain placement may suffice. However, there is a need for further research to improve the suspicion and diagnosis of Meckel’s diverticulum perforations in neonates. Additionally, vigilant assessment and timely intervention for adhesive intestinal obstruction can enhance bowel salvage outcomes.

## Introduction

Pneumoperitoneum, the presence of air within the abdominal cavity, is considered a surgical emergency in neonates. Necrotizing enterocolitis is typically the most common cause, although other factors, such as perforations resulting from conditions like imperforate anus, Hirschsprung’s disease, small bowel atresia, meconium ileus, gastric overinflation or inflammation, and spontaneous intestinal perforation, also contribute [1,2]. Perforated Meckel’s diverticulum, although extremely rare in neonates, has been reported in the literature, with only 59 cases identified since 1928, most of which were diagnosed through exploratory laparotomy or autopsy [3]. Before treating pneumoperitoneum, weighing the risks and benefits of available interventions is critical. Traditional teaching supports the success of initial drain placement and primary laparotomy in specific instances. This case illustrates the effectiveness of both strategies in appropriately selected patients. The development of adhesions leading to mechanical bowel obstruction is a common outcome following abdominal surgery, with incidences in small children ranging from 1% to 13%, especially within the first six months following the procedure [4]. Despite the inability to prevent this issue entirely, we explored the literature for potential preventive measures. This report details the case of a preterm, extremely low birth weight infant who experienced pneumoperitoneum due to a perforated Meckel’s diverticulum, followed by adhesive intestinal obstruction.

## Case presentation

**Figure 1:**
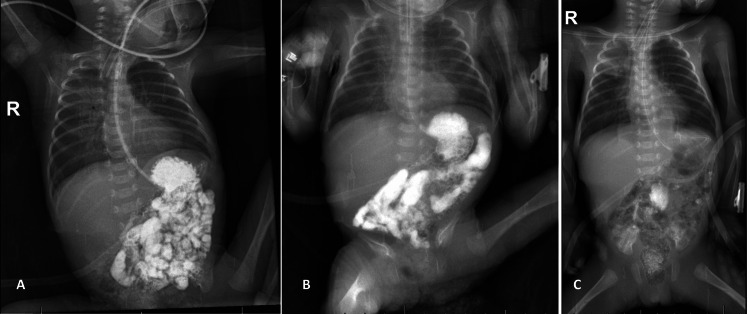
Sequential X-ray images with oral contrast medium showing the progression of contrast through the gastrointestinal tract The series includes images at (A) two hours, (B) six hours, and (C) 24 hours after ingestion, demonstrating unobstructed flow into the rectum and beyond without any spillage or obstruction.

A female infant was delivered spontaneously at 29 weeks gestation in our hospital and was admitted to the neonatal intensive care unit. Her birth weight was 850 grams. The mother experienced sudden labor pains for an unknown reason, although her antenatal history was otherwise unremarkable. On the sixth day of life, we were consulted for pneumoperitoneum detected on a lateral decubitus cross-table abdominal X-ray. The infant’s symptoms included gradually increasing abdominal distention, though she was passing normal stools and had received small trophic feeds the day before. Upon examination, no signs of peritonitis were present. Laboratory test results were within reference ranges except for a decrease in platelet count to 125 x 10^9/L. Given her young age, weight of less than 1 kg, and absence of peritonism, we opted to insert a peritoneal drain at the bedside, which only released a gush of air. We initiated comprehensive supportive care, including broad-spectrum intravenous antibiotics and parenteral nutrition. The drain remained dry, and the infant’s abdominal condition and physiological state improved. X-ray imaging with oral isotonic water soluble normoosmolar contrast, performed 14 days after drain insertion, demonstrated an unobstructed passage to the rectum, no intraperitoneal spillage, and no strictures (Figure 1).

**Figure 2:**
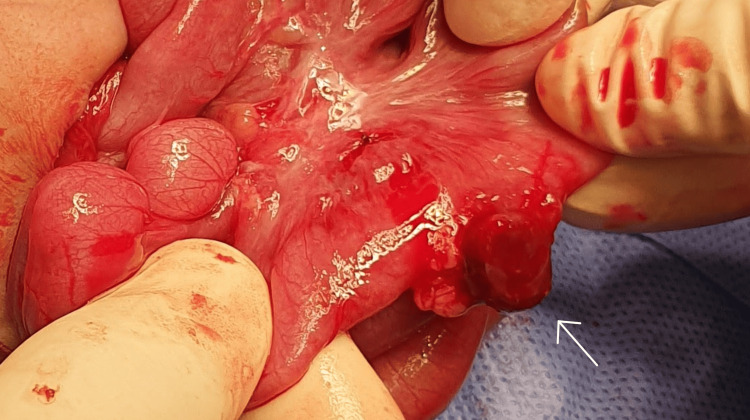
Perioperative photograph illustrating a perforated Meckel’s diverticulum with a visible raw surface (arrow), identified following adhesiolysis

**Figure 3:**
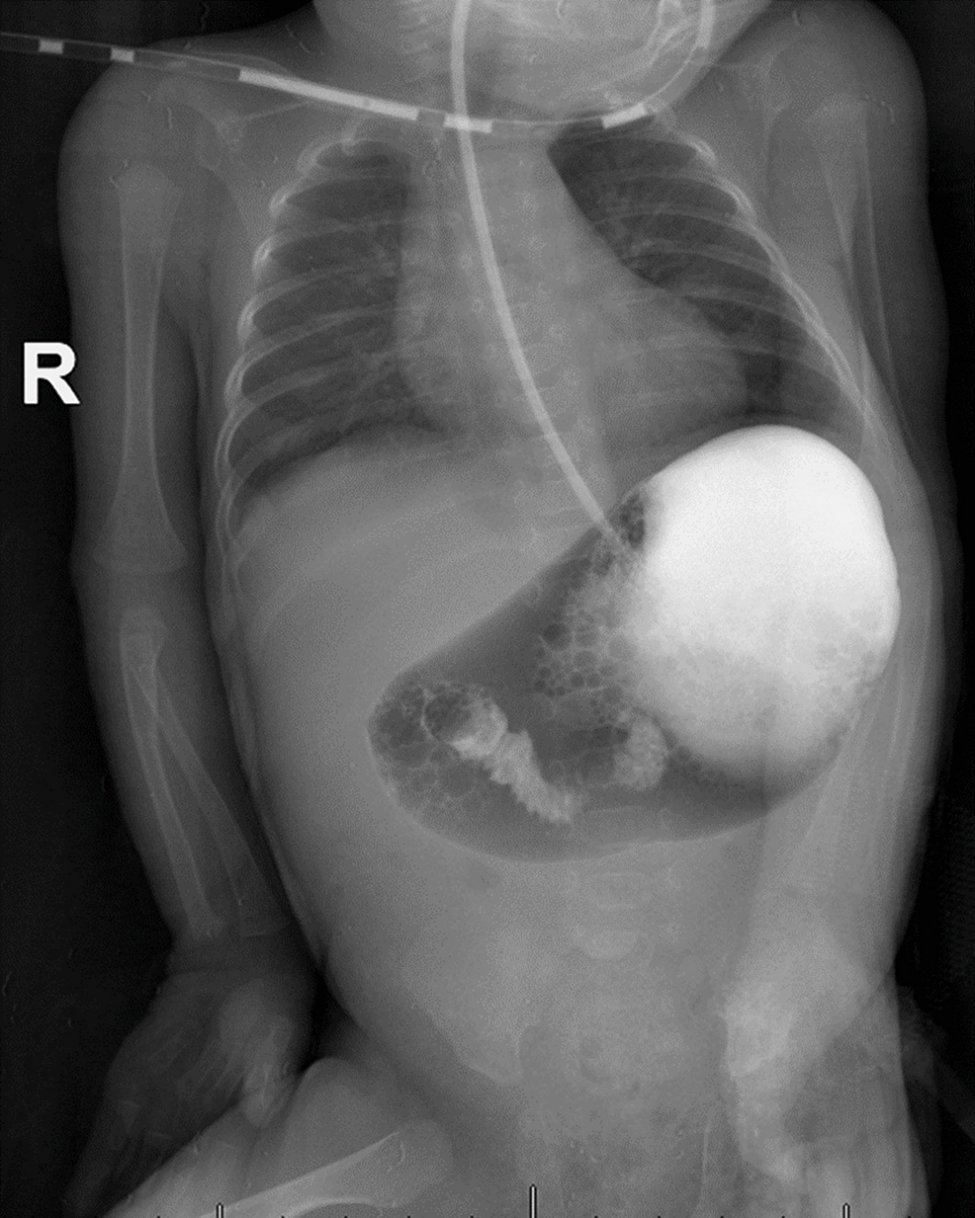
X-ray imaging with contrast medium revealing complete bowel obstruction, highlighting the severity of the condition

The drain was removed that day, and the infant gradually resumed feeding, eventually achieving full feeds. Fourteen days after the drain’s removal, she again developed abdominal distention, and a lateral decubitus cross-table abdominal X-ray confirmed pneumoperitoneum. Her weight had increased to 1.10 kg, and her corrected gestational age was 34 weeks. An exploratory laparotomy revealed a perforated Meckel’s diverticulum (Figure 2). We resected the bowel a few millimeters on either side and performed an end-to-end, single-layer extramucosal anastomosis. We also addressed several adhesions within the peritoneal cavity, removed them, and washed the abdomen with warmed saline. The patient recovered well postoperatively and was discharged upon achieving full feeding. One month and 20 days post-surgery, during the third neonatal intensive care unit consultation, the corrected age was 41 weeks, and her weight had reached 1.7 kg. Despite a bilious aspirate, an abdominal X-ray was inconclusive for obstruction. Serial X-ray imaging with oral contrast medium did not reveal passage beyond the proximal jejunum 12 hours after administration (Figure 3), raising concerns for ischemia due to a closed-loop bowel obstruction.

A subsequent laparotomy revealed extensive interloop adhesions and band obstruction, including a closed loop. After releasing the band and performing adhesiolysis, we confirmed the bowel’s patency from the duodenojejunal junction to the ileocecal junction. The patient made a significant recovery post-surgery. At the eight-month follow-up, the child remained asymptomatic and well.

## Discussion

In managing pneumoperitoneum in neonates, using an intraperitoneal drain, while still debated, allowed our patient to resume feeding after the first episode. Inserting the drain when the baby weighed 900 grams provided critical time to stabilize her condition and enhance her nutritional status. The baby gained 200 grams, reaching a weight of 1.1 kg, before requiring surgical intervention for a second pneumoperitoneum episode. It is important to note that a primary laparotomy does not preclude the possibility of future surgeries. Research indicates that initial laparotomy may necessitate a second surgery due to complications, such as adhesive intestinal obstruction, or for stoma reversal [5]. However, the same studies also highlight a significant recurrence of pneumoperitoneum in cases initially managed with only an intraperitoneal drain.

Managing spontaneous intestinal perforation in premature infants with a peritoneal drain alone is considered a safe approach [5-7]. In our patient, the perforation sealed spontaneously without causing any complications, a phenomenon documented in children, including neonates [8,9]. Initially, we suspected that the spontaneous intestinal perforation had sealed by itself. However, we later identified the cause as a self-sealed perforated Meckel’s diverticulum, which was evident from the per-operative findings. Although the literature reports a 91% closure rate of fistulas within four weeks in adults [10], our case demonstrated sealing within two weeks, underscoring the remarkable healing capacity of infants and the potential for conservative management in selected cases.

This case underscores the necessity of developing strategies to diagnose or suspect Meckel’s diverticulum. Despite the rarity of perforated Meckel’s diverticulum in neonates, with only 59 cases reported since 1928 and seven in premature infants [3,11], its presentation can vary widely, including symptoms such as abdominal distention, bilious vomiting, reduced bowel sounds, and respiratory distress, all of which are nonspecific. Although nuclear scanning is a diagnostic tool for this condition, its results can be inconsistent due to factors like scan timing, pre-medication effects, and patient demographics [12]. A high index of suspicion is warranted in cases presenting with a sudden release of air upon drain insertion that recovers without exploratory laparotomy.

Our histopathological examination revealed no gastric or pancreatic tissue in the resected specimen, indicating that their presence is not a prerequisite for Meckel’s diverticulum perforation. This finding suggests that proton pump inhibitors may not be beneficial in such cases and challenges the decision to leave an incidental Meckel’s diverticulum untreated. Current literature suggests removal if certain risk factors are present: age under 50 years, male sex, diverticulum length over 2 cm, and abnormal features within the diverticulum [13]. We propose that incidental Meckel’s diverticulum in premature infants should be carefully considered for removal based on these criteria, warranting further research.

Despite meticulous washing during the second laparotomy, the infant developed adhesive intestinal obstruction, the cause of which remained unclear. Our prompt intervention prevented ischemia and bowel resection, illustrating the benefits of early exploration. The management of adhesive intestinal obstruction varies widely, with some surgeons adopting a conservative approach and others opting for early surgical intervention. Both operative and nonoperative treatments are accepted standards influenced by patient factors, available resources, surgeon experience, and institutional practices [14]. Extensive research into pharmacological agents to prevent adhesion formation is ongoing, focusing on lubricants, antifibrotic, and anti-inflammatory agents, though human trials are pending. The use of barrier agents like INTERCEED® (Ethicon, Somerville, NJ, US) and Seprafilm (Baxter International Inc., Deerfield, IL, US) has been associated with an increased risk of anastomotic leak when placed around new anastomoses and suture lines [15]. Preventative strategies emphasize minimizing injury to serosal and parietal peritoneal surfaces [16].

## Conclusions

Our case demonstrates that inserting a peritoneal drain in neonates who are not suitable candidates for surgery due to their age or weight poses no harm. Clinicians should maintain a high level of suspicion for perforated Meckel’s diverticulum, particularly in instances where only a rush of air is observed upon drain insertion, followed by clinical improvement. Evaluating and managing adhesive intestinal obstruction in neonates requires careful consideration, as standardized guidelines are not yet established. A vigilant approach and prompt surgical exploration are essential to prevent bowel ischemia, thereby enhancing the child’s quality of life.
